# Early warnings of the potential for malaria transmission in rural Africa using the hydrology, entomology and malaria transmission simulator (HYDREMATS)

**DOI:** 10.1186/1475-2875-9-323

**Published:** 2010-11-12

**Authors:** Teresa K Yamana, Elfatih AB Eltahir

**Affiliations:** 1 Department of Civil and Environmental Engineering, Massachusetts Institute of Technology, 77 Massachusetts Avenue, Cambridge, MA 02139, USA

## Abstract

**Background:**

Early warnings of malaria transmission allow health officials to better prepare for future epidemics. Monitoring rainfall is recognized as an important part of malaria early warning systems. The Hydrology, Entomology and Malaria Simulator (HYDREMATS) is a mechanistic model that relates rainfall to malaria transmission, and could be used to provide early warnings of malaria epidemics.

**Methods:**

HYDREMATS is used to make predictions of mosquito populations and vectorial capacity for 2005, 2006, and 2007 in Banizoumbou village in western Niger. HYDREMATS is forced by observed rainfall, followed by a rainfall prediction based on the seasonal mean rainfall for a period two or four weeks into the future.

**Results:**

Predictions made using this method provided reasonable estimates of mosquito populations and vectorial capacity, two to four weeks in advance. The predictions were significantly improved compared to those made when HYDREMATS was forced with seasonal mean rainfall alone.

**Conclusions:**

HYDREMATS can be used to make reasonable predictions of mosquito populations and vectorial capacity, and provide early warnings of the potential for malaria epidemics in Africa.

## Background

The Roll Back Malaria (RBM) initiative has published a framework for malaria early warning systems (MEWS) in Africa [1]. These systems rely on indicators of vulnerability, transmission risk and early case detection in order to predict the onset and severity of malaria epidemics. Monitoring rainfall has been recognized as an essential component for MEWS and is being used by malaria control programmes in a number of African countries [2]. Hay *et al *[3] retrospectively determined that monitoring dekadal (every 10 days) estimates of rainfall anomalies provided by the Africa Data Dissemination Service (ADDS) could have provided a reliable warning of a major malaria epidemic that occurred in 2002 in Kenya. Thomson *et al *[4] suggested that in Botswana, rainfall from December through February could be used to give an early warning for high transmission years.

While excess rainfall is often associated with increased malaria transmission, this is not always the case. For example, heavy rainfall associated with the 1997-98 El Nino event was associated with decreased malaria transmission in the highlands of Tanzania, presumably by washing away larval breeding sites [5]. Similarly, decreases in rainfall have been observed to increase malaria transmission by creating breeding pools in areas where flowing water would normally wash larva away [6]. With a hydrology driven model such as Hydrology, Entomology and Malaria Transmission Simulator (HYDREMATS), the relationships between anomalous levels of rainfall and malaria transmission can be explicitly represented, allowing the user to draw the correct conclusions from information regarding rainfall patterns.

The development of HYDREMATS is described in detail in Bomblies *et al *[7]. The model was developed to simulate village-scale response of malaria transmission to interannual climate variability in semi-arid desert fringe environments such as the Sahel. The model provides explicit representation of the spatial determinants of malaria transmission. HYDREMATS can be separated into two components: the hydrology component which explicitly represents pooled water available to anopheles mosquitoes as breeding sites, and the entomology component, which is an agent-based model of disease transmission.

In the hydrology component, rainfall is partitioned between runoff and infiltration, with soil and vegetation properties strongly influencing the partition between these two processes. Uptake of soil water from evapotranspiration is calculated based on climatic variables. Overland flow is modelled using a finite difference solution, and flow velocity is calculated as a function of friction slope, flow depth, and a distributed roughness parameter derived from soil characteristics and vegetation type. The overland flow process is of critical importance for the modelling of water pool formation. The hydrology component of HYDREMATS simulates the spatial distribution of water depths and temperatures for each grid cell, for each timestep. These distributions serve as the inputs for the entomology component of the model [7].

The entomology component of HYDREMATS simulates individual mosquito and human agents. Human agents are immobile, and are assigned to village residences, as malaria transmission in this region occurs primarily at night when humans are indoors [8]. Mosquito agents have a probabilistic response to their environment based on a prescribed set of rules governing dispersal and discrete events including development of larval stages, feeding, egg-laying and death. The model tracks the location, infective status and reproductive status of each female mosquito through time. Mosquitoes become infected when they bite an infectious human, and after a temperature dependant time lag, can transmit the parasite to humans during subsequent blood meals [7].

In addition to the water depth inputs supplied by the hydrology component of the model, the entomology component requires air temperature, humidity, wind speed and wind direction. Air temperature and relative humidity influence mosquito behaviour and survival, while wind speed and direction influence mosquito flight, both by physical displacement by wind, and by attracting mosquitoes to upwind blood sources. The location of village residences is required in order to assign the location of human agents [7]. The outputs from HYDREMATS include the number of adult mosquitoes and the vectorial capacity at each time step. Vectorial capacity is a measure of the mosquito's ability to transmit disease, and is defined as the average number of human inoculations of a parasite originating from a single case of malaria, if all vectors biting the original case were to become infected [9].

There is a natural lag time between rainfall and malaria transmission, as rainfall must first be routed into water pools, and eggs laid in these pools must develop to adulthood before they can begin transmitting the disease. In this study, a simple method was developed to estimate rainfall two and four weeks in advance. The lead time gained by these forecasts combined with the natural lag time allows us to use HYDREMATS to make accurate predictions for the potential for malaria transmission as measured by vectorial capacity several weeks in advance. While this does not have the same advantages of a warning several months in advance, it has less uncertainty than longer range seasonal forecasts, and it could nonetheless be helpful as it would allow malaria control programmes some time to redistribute drug supplies, prepare health clinics for an influx of cases, engage in vector control activities and raise public awareness.

## Methods

### Study location

**Figure 1:**
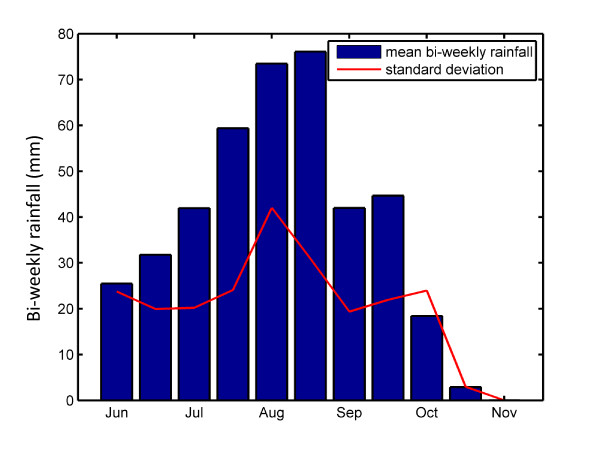
**Seasonal rainfall in Banizoumbou, Niger 1997-2008**. The mean total rainfall for each two-week period is shown in blue, and the standard deviation of bi-weekly rainfall is shown in red.

The simulations described here were conducted over the domain of Banizoumbou village in south-western Niger. Banizoumbou is a typical Sahelian village in a semi-arid landscape, with a population of about 1000. Mean bi-weekly rainfall in Banizoumbou was calculated using the Global Daily Merged Precipitation Analyses of the Global Precipitation Climatology Project (GPCP) [10]. GPCP values from 1997 to 2008 were averaged for each two-week period between June 1^st ^and October 31^st^. Although a longer time-record would be desirable, GPCP with bi-weekly resolution was only available over these 11 years. The mean and standard deviation of biweekly precipitation totals over the 12-year period is shown in Figure 1. The graph shows a strong seasonality of rainfall, with a peak in August. During this time, water pools form in and around the village, providing ideal breeding habitat for *Anopheles gambiae *mosquitoes. Mosquito populations and malaria transmission in Niger increase dramatically during the rainy season [7].

Mosquito density data were collected in Banizoumbou. Meteorological data for Banizoumbou are available through the African Monsoon Multidisciplinary Analyses (AMMA) database.

### Construction of predicted rainfall time series

When attempting to predict rainfall with a lead time on the order of two weeks, the important factors to consider are the seasonality, the history, and the persistence of rainfall. An analysis of the rainfall anomalies during each two-week period over the 12 years showed that there was very little correlation between the anomalies of two consecutive two-week periods (see Additional file 1: Standardized anomalies of adjacent biweekly precipitation totals). Since there was little persistence in rainfall patterns on the two-week time-scale, the seasonality and the history of rainfall were used to make predictions of rainfall into the future.

**Figure 2:**
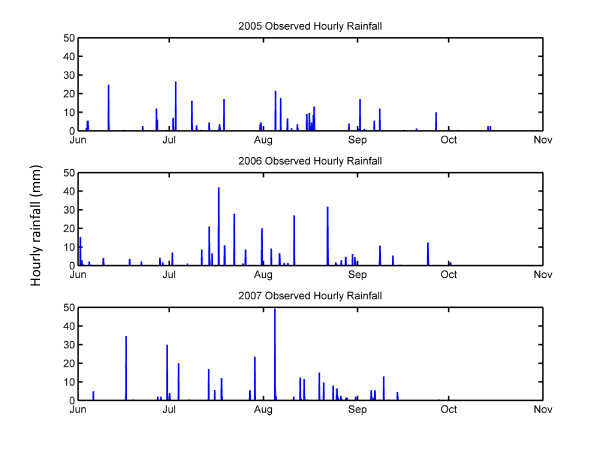
**Hourly rainfall in Banizoumbou, Niger**. Hourly rainfall measured by a rain gauge in Banizoumbou village during the rainy seasons of 2005, 2006 and 2007.

This investigation focused on Banizoumbou village during the rainy seasons of 2005, 2006 and 2007. Hourly rainfall measured by a rain gauge for this time period is shown in Figure 2. HYDREMATS simulations using observed rainfall were conducted and taken as control simulations. The observed sequence of rainfall is referred to as pattern 1.

**Figure 3:**
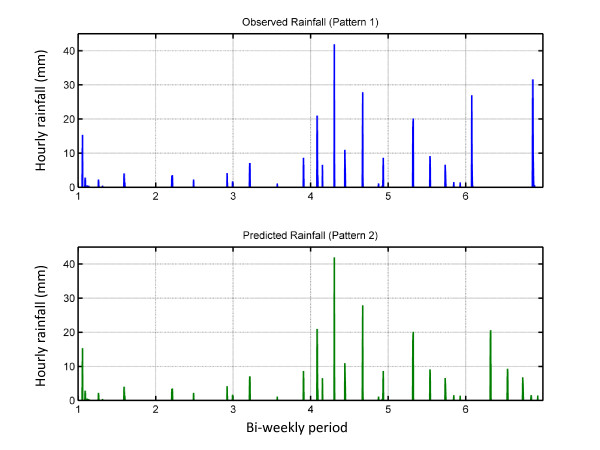
**Rainfall Inputs for the 6th Bi-weekly Period of the 2006 rainy season**. The first plot shows observed hourly rainfall. The second graph shows the predicted rainfall series. This series uses observed rainfall from the first five bi-weekly period, followed by a prediction for the following two weeks, made by repeating the pattern of rainfall observed during the fifth bi-weekly period, scaled such that the total volume is equal to the mean volume for that period.

The prediction rainfall data sets were created by taking the observed rainfall up until the time of the prediction, thus incorporating the history of the system. In order to forecast the rainfall sequence and magnitude for the future two weeks, the actual rainfall time series for the latest two weeks was scaled such that the total volume of rainfall was equal to the mean of historically observed rainfall corresponding to the future two-week period based on the GCPC data. This method incorporates the information about seasonality, while distributing rainfall in a realistic manner. A rainfall time series was created in this manner starting on June 1^st ^and continuing to each successive two-week period until October 31^st ^for 2005, 2006 and 2007. This pattern of rainfall is defined as pattern 2. Figure 3 shows rainfall inputs for simulations for the sixth bi-weekly period of the 2006 rainy season, which corresponds to late-August. The first plot shows observed rainfall. The second graph shows the predicted rainfall series. This series uses observed rainfall from the first five bi-weekly periods. The pattern of rainfall observed for the fifth period is repeated in the sixth period, scaled such that the total volume is equal to the mean volume for that period, which was 76 mm. This same procedure was repeated to produce rainfall time series with predictions extending to four weeks into the future.

### Simulations

A HYDREMATS simulation was conducted for each of the predicted rainfall series. In order to isolate the effects on malaria forecasts based on rainfall inputs, real time measurements of the remaining environmental inputs to HYDREMATS, which are temperature, relative humidity, radiation, wind speed and wind direction, were used in all simulations. In a true prediction scenario, the future values of these variables would need to be estimated as well. This can be done by extending the method used here for rainfall to include other environmental variables, or by using predictions from medium range weather forecasting or seasonal forecasting systems.

In order to examine the benefit of including the history of the magnitude of observed rainfall in the prediction, a new set of rainfall inputs was created by scaling the pattern 2 rainfall prediction series such that the total volume of rainfall in each two-week period was equal to the seasonal mean for that period. These rainfall inputs are referred to as 'seasonal mean (pattern 2)'. Simulations forced by seasonal mean (pattern 2) rainfall are predictions for the following two weeks, made without the benefit of knowing the magnitude of past rainfall.

## Results

### Predictions made 2 weeks in advance

**Figure 4:**
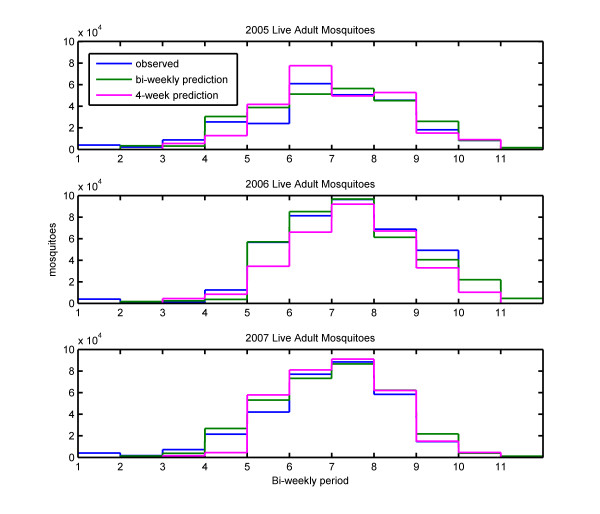
**Simulated adult mosquito population**. Bi-weekly mean mosquito populations for the observed rainfall simulation in blue, the prediction made two weeks in advance in green, and the prediction made four weeks in advance in pink.

**Figure 5:**
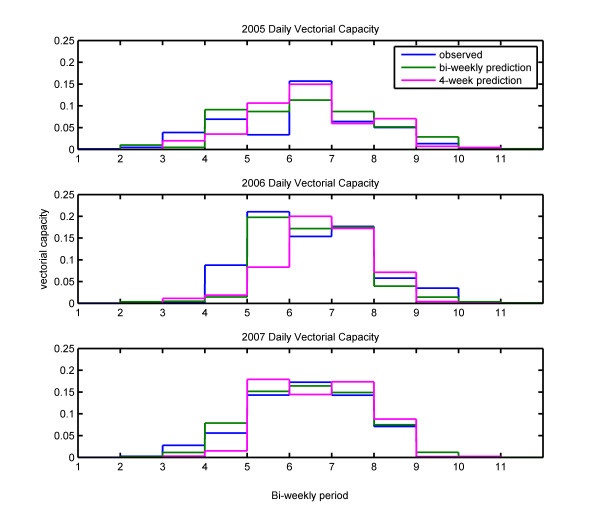
**Simulated vectorial capacity**. Bi-weekly mean vectorial capacity for the observed rainfall simulation in blue, the prediction made two weeks in advance in green, and the prediction made four weeks in advance in pink.

The accuracy of this prediction method can be assessed by comparing results from the simulations using observed and predicted rainfall, shown in Figures 4 and 5. These figures show mosquito populations and vectorial capacity respectively, with the bi-weekly mean mosquito populations for the observed rainfall simulation in blue, the prediction made two weeks in advance in green, and the prediction made four weeks in advance in pink. The numbers on the x-axis denote the times at which the predictions for the following two or four weeks were made, with 1 corresponding to June 1^st^, and each subsequent mark corresponding to an additional two weeks. Bi-weekly predictions were made at each interval, while four-week predictions were made only at the odd numbered intervals.

The results shown in Figures 4 and 5 demonstrate that this simple method of predicting rainfall leads to a prediction of mosquito populations that is quite accurate. In all three years, the predicted number of mosquitoes follows the same seasonal cycle as the number of mosquitoes in the simulation using observed rainfall. This method correctly predicted that the peak number of mosquitoes in 2006 and 2007 would be significantly greater than the peak in 2005. The timing of the peak was also correctly predicted in 2006 and 2007, but was predicted to be two weeks later than observed in 2005.

In all three years, the period of peak transmission was correctly identified. Vectorial capacity was overestimated for the fourth and fifth bi-weekly periods of 2005, and underestimated during the sixth period, which was when peak transmission occurred. Vectorial capacity for the fourth bi-weekly period of 2006 was underestimated. Otherwise, predicted values of vectorial capacity were very close to the values obtained when the model was forced with observed rainfall.

### Predictions made four weeks in advance

Although the predictions were made every four weeks, the results are presented in Figures 4 and 5 as two two-week estimates in order to maintain a two-week resolution for mosquito populations and vectorial capacity. Comparing mosquito populations from the observed rainfall simulations to the four-week prediction simulations shows that the prediction simulations were able to forecast the seasonal pattern of mosquito populations. However, in most cases, estimates made four weeks in advance resulted in estimates further from the control than estimates made only two weeks in advance. In general, the four-week predictions made for the second half of the malaria season were very close to the control, while predictions for the early part of the season were less consistent. The predictions for vectorial capacity made four weeks in advance, were also generally less accurate than estimates made two weeks in advance, with estimates for the second half of the season being significantly more accurate than estimates for the early months of the rainy season. These findings suggest that a reasonable prediction method would be to make predictions with two-week lead time for the early rainy season, transitioning to four-week predictions in the second half of the season.

### The benefit of using the history of rainfall in a prediction scenario

**Figure 6:**
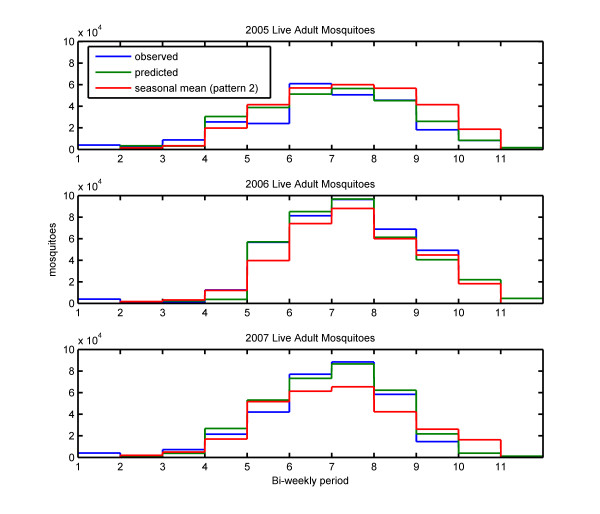
**Simulated adult mosquito population**. Mean number of live female adult mosquitoes for each two-week period during the rainy seasons of 2005, 2006 and 2007 with the simulations forced by observed rainfall (pattern 1) in blue, 2-week prediction rainfall series (pattern 2) in green, and seasonal mean (pattern 2) rainfall series in red.

Figure 6 shows the mean number of live female adult mosquitoes for each two-week period during the rainy seasons of 2005, 2006 and 2007 with the simulations forced by observed rainfall (pattern 1) in blue, 2-week prediction rainfall series (pattern 2) in green, and seasonal mean (pattern 2) rainfall series in red. The value of including the history of rainfall in the prediction can be assessed by comparing results from the prediction simulation and the seasonal mean (pattern 2) simulation.

It is apparent that for predicting mosquito populations, the history of rainfall is important, as the mean number of mosquitoes estimated by the prediction simulations were almost always closer to the observed rainfall simulations than the seasonal mean simulations were. This is most evident in the middle of the rainy season in 2007, where the seasonal mean simulation significantly underestimated the peak mosquito populations occurring in the sixth through ninth bi-weekly periods. Total rainfall in 2005 was close to the annual mean total, so the difference between the prediction and the seasonal mean simulations is less apparent. However, a consistent over-estimation of mosquito populations is observed in the later part of 2005 in the seasonal mean simulation, while the prediction simulation was very close to the observed rainfall simulation. The seasonal mean simulation underestimated mosquito populations in the fifth through ninth bi-weekly periods in 2006, which is the period of peak number of mosquitoes; estimates given by the prediction simulation during this period were closer to the values obtained in the observed rainfall simulation.

**Figure 7:**
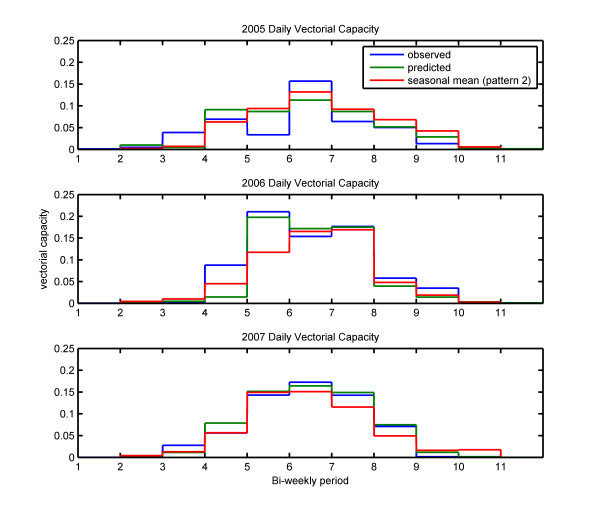
**Simulated vectorial capacity**. Mean vectorial capacity for each two-week period during the rainy seasons of 2005, 2006 and 2007 with the simulations forced by observed rainfall (pattern 1) in blue, 2-week prediction rainfall series (pattern 2) in green, and seasonal mean (pattern 2) rainfall series in red.

Interestingly, the advantage of using the history of rainfall magnitude is much less apparent in the vectorial capacity results, shown in Figure 7. The seasonal mean simulations produced estimates of vectorial capacity comparable to those produced by the prediction simulations. The seasonal mean simulation in 2006 did not predict the sharp increase in vectorial capacity in the fifth bi-weekly period, while this signal was successfully reproduced in the prediction simulation. Vectorial capacity was consistently underestimated in the sixth through eight bi-weekly periods of 2007 in the seasonal mean scenario, while the prediction simulation for this time period resulted in vectorial capacity estimates very close to the values obtained by the observed rainfall simulation.

### Comparison with field observations

The validity of the simulations and predictions can be assessed by comparing outputs to field observations. While it is impossible to quantify the absolute number of mosquitoes over the model domain, trends in mosquito abundance are reflected in trends in captured mosquitoes. During the rainy seasons of 2005 and 2006, six CDC miniature light traps were deployed in Banizoumbou, four inside residences, and two outdoors. Sampling occurred weekly, from 7 p.m. to 7 a.m. At the end of the sampling period, all mosquitoes were removed, and *Anopheles gambiae sensu lato *mosquitoes were identified and counted [7]. The mosquito capture data had a pronounced dependence on lunar phase, as has been noted by numerous studies (e.g., [11,12]). This occurs because moonlight competes with bulbs from light traps as a mosquito attractant; the bulb attracts mosquitoes from greater distances on moonless nights than on the brightly lit nights of a full moon. In order to correct for this bias, the number of captured mosquitoes at each sampling time were divided by the effective capture area of the trap for that night. The resulting mosquito densities are assumed to be independent of lunar phase [7].

**Figure 8:**
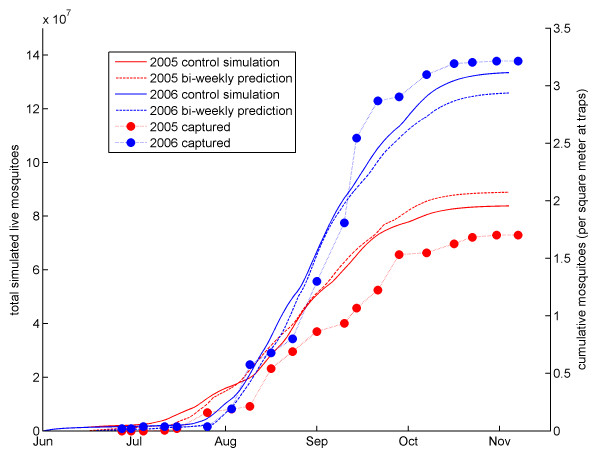
**Comparison with field observation**. 2005 data are shown in red, and 2006 data are shown in blue. The solid line represents adult mosquitoes modelled in the control simulation, the dashed line shows adult mosquitoes in the bi-weekly prediction simulation. The circles show mosquitoes captured in the field, corrected for moon phase.

Cumulative simulated mosquitoes and cumulative density of captured mosquitoes are shown in Figure 8. In this figure, simulated mosquitoes are shown on the left axis, with the solid lines corresponding to the simulations using observed rainfall, and the dashed lines corresponding to the bi-weekly prediction simulations. Mosquito densities are shown on the right axis. Data from 2005 are shown in red, and data from 2006 are shown in blue. This figure shows that simulations using both observed and predicted rainfall resulted in roughly 1.5 times as many cumulative mosquitoes in 2006 than in 2005. This dramatic increase in simulated mosquito numbers is consistent with the observed mosquito densities. While the cumulative mosquitoes from the prediction simulations are further from the field observations than the control simulations for both, the predicted relative difference between 2005 and 2006 is clearly pronounced. This finding gives us confidence that HYDREMATS can serve as a reliable predictor of relative mosquito abundance and hence the potential for malaria transmission.

## Discussion

The results of this analysis showed that HYDREMATS is effective at making short-term predictions of mosquito populations and vectorial capacity by using a simple method of predicting future rainfall, combined with known information on past rainfall. By mechanistically linking rainfall to malaria transmission, HYDREMATS offers an advantage over the direct monitoring of rainfall or the use of statistical relationships between rainfall and malaria. HYDREMATS uses rainfall inputs to simulate the hydrologic processes by which pools are formed, and the biological processes by which these pools lead to increased numbers of mosquitoes by serving as larval habitats. HYDREMATS takes into account details such as short term rainfall patterns and the location of water pools relative to households that are overlooked by methods considering only aggregated rainfall totals.

HYDREMATS was able to replicate the seasonal cycle and inter-annual variations of both mosquito populations and vectorial capacities two and four weeks in advance. This could prove extremely useful to malaria control programs by giving an advance warning of periods with higher than expected malaria transmission, allowing health officials to prevent or prepare for an oncoming epidemic by engaging in activities such as redistributing limited medical staff and supplies, preparing health workers for prompt case detection and treatment, conducting vector control activities, and raising public awareness of heightened risk of malaria transmission.

The history of observed rainfall was shown to be important in making an accurate prediction of mosquito populations, but somewhat less important in the prediction of vectorial capacity. This means that some of the information used by HYDREMATS in predicting mosquito populations is based on rainfall that is already known to have occurred, which increases the accuracy of the prediction, compared to a prediction based entirely on predicted future rainfall.

The cumulative number of simulated mosquitoes was significantly higher in 2006 than in 2005 for both the control simulations and the predictions. This relative difference in abundance is consistent with field observations of adult mosquitoes. This finding indicates that HYDREMATS can serve as a reliable simulator and predictor of mosquito abundance.

## Conclusions

A method has been demonstrated by which mechanistic modelling of hydrological and entomological processes can be used to make short-term predictions of mosquito populations and malaria transmission. This method is an improvement over methods based on direct monitoring of rainfall or statistical correlations relating rainfall to malaria transmissions, as it explicitly represents the mechanistic relationships between observed rainfall, mosquito populations, and the subsequent response in malaria transmission.

## Competing interests

The authors declare that they have no competing interests.

## Authors' contributions

TKY conducted the modelling study and drafted the manuscript. EABE supervised the study, provided advice for study design and implementation, and participated in writing the final version of the manuscript. All authors read and approved the final manuscript.

## Supplementary Material

Additional File 1 **Standardized anomalies of adjacent biweekly precipitation totals**. To determine the extent of persistence of rainfall, the standardized anomaly of rainfall, which is defined as the departure from the seasonal mean divided by the standard deviation for that time period, during each two-week period in Banizoumbou between 1997 and 2008 was calculated, and compared to that of the following two-week period. This analysis, shown in Additional File 1, showed that there was very little persistence of rainfall amounts between two-week periods, with a correlation coefficient of only 0.07. Since there was little persistence in rainfall patterns on the two-week time-scale, the history and seasonality of rainfall were used to make predictions of rainfall 14 days into the future. Click here for file
