# Supplementary figures and images for: Early warnings of the potential for malaria transmission in rural Africa using the hydrology, entomology and malaria transmission simulator (HYDREMATS)
Source: Malar J. 2010 Nov 12;9:323. doi: 10.1186/1475-2875-9-323 (PMC2989331; doi:10.1186/1475-2875-9-323)

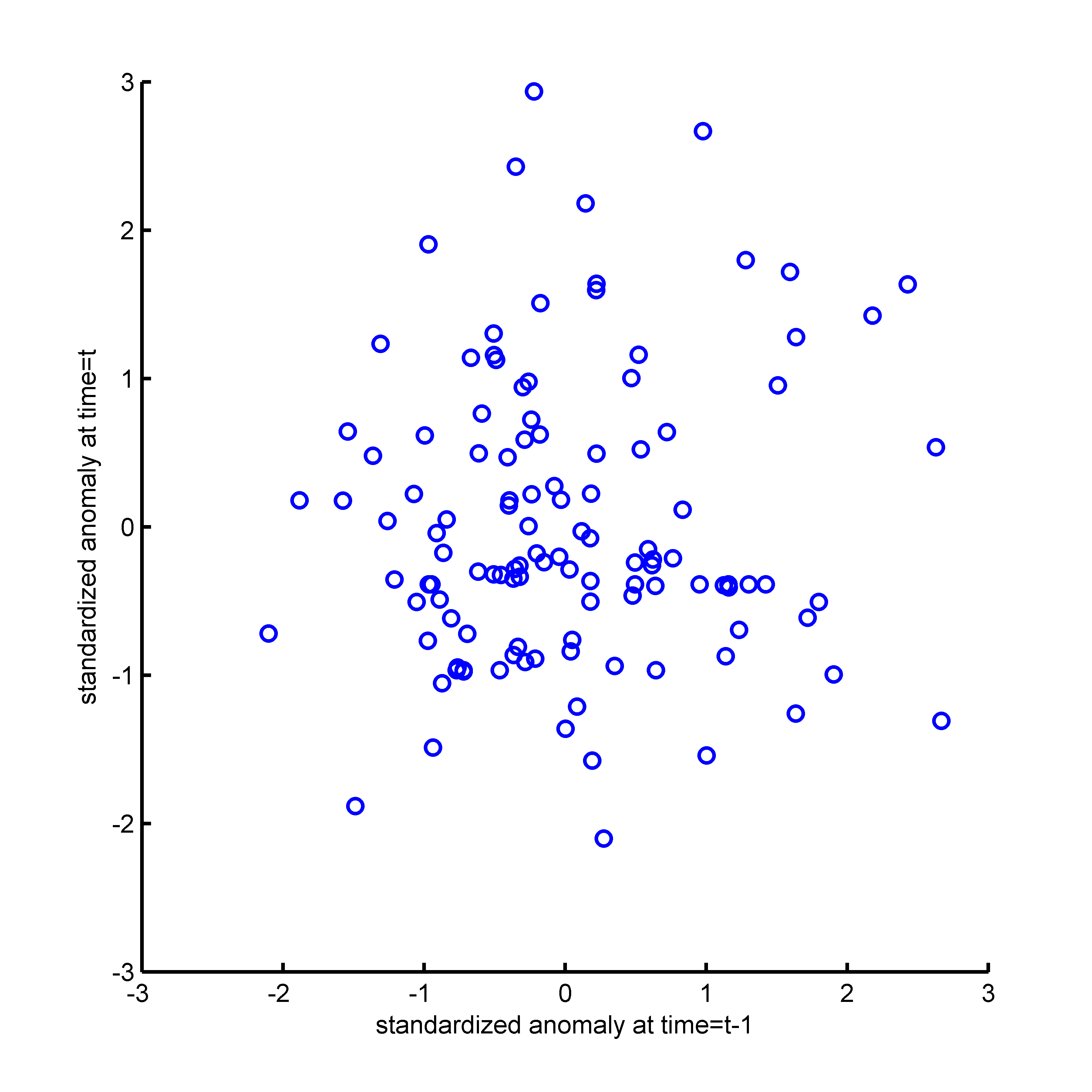

Supplement: Additional File 1 — Standardized anomalies of adjacent biweekly precipitation totals. To determine the extent of persistence of rainfall, the standardized anomaly of rainfall, which is defined as the departure from the seasonal mean divided by the standard deviation for that time period, during each two-week period in Banizoumbou between 1997 and 2008 was calculated, and compared to that of the following two-week period. This analysis, shown in Additional File 1, showed that there was very little persistence of rainfall amounts between two-week periods, with a correlation coefficient of only 0.07. Since there was little persistence in rainfall patterns on the two-week time-scale, the history and seasonality of rainfall were used to make predictions of rainfall 14 days into the future. [file 1475-2875-9-323-S1.TIFF]
